# Autosomal dominant polycystic kidney disease and minimal trauma: medical review and case report

**DOI:** 10.1186/s12873-018-0192-3

**Published:** 2018-11-01

**Authors:** Karim Hajjar, Ralphe Bou Chebl, Mohammad Kanso, Gilbert Abou Dagher

**Affiliations:** Department of Emergency Medicine, American University of Beirut Medical Center, P.O. Box 11-0236, Riad El-Solh, Beirut, 1107 2020 Lebanon

**Keywords:** Hemorrhage, Polycystic kidney disease, Renal injury, Trauma

## Abstract

**Background:**

Blunt abdominal trauma in the setting of polycystic kidney disease is still scantly described in the literature and management guidelines of such patients are not well-established.

**Case presentation:**

The authors herein present a case of hypovolemic shock secondary to segmental renal artery bleed in a 75-year-old man with polycystic kidney disease after minimal blunt abdominal trauma, who underwent successful selective arterial embolization, and provide a thorough review of similar cases in the literature, while shedding the light on important considerations when dealing with such patients.

**Conclusions:**

It is important to suspect renal injury in patients with pre-existing renal lesions irrespective of the mechanism of injury; and, vice-versa to suspect an underlying abnormality in patients with a clinical deterioration that’s out of proportion to the mechanism of injury.

**Electronic supplementary material:**

The online version of this article (10.1186/s12873-018-0192-3) contains supplementary material, which is available to authorized users.

## Background

The kidney is the most frequently injured genitourinary organ, with a female to male ratio of 1:3 [1]. Renal injuries occur in up to 1 patient for every 20 trauma cases and are categorized into blunt and penetrating [2]. Overall, most kidney injuries stem from blunt trauma and tend to be generally less severe, with less nephrectomy rates as compared to penetrating injuries [3]. Common mechanisms of injury include motor vehicle crashes, lower rib fractures, falls and direct blows. Additionally, significant decelerating forces may result in avulsion of the renal pedicle or dissection of the renal artery. The kidneys are located in the retroperitoneal space and are protected by the back musculature, the lower ribs, and the perinephric fat, thus, significant force and major trauma is usually required to injure the kidney [4, 5]. However, in the presence of pre-existing renal lesions, trivial trauma can result in blunt renal injury and ensuing symptoms can get out of proportion to the type of injury in these cases [6]. This topic is scantly described in the literature and management guidelines of such patients are still lacking.

We report on the case of a patient with ADPKD who presented with shock to the Emergency Department secondary to trivial blunt abdominal trauma. In addition, we provide a review of patients with Autosomal Dominant Polycystic Kidney Disease (ADPKD) who sustain a kidney injury following trauma.

### Objectives

The objective of this review is to describe a case of hypovolemic shock secondary to minimal blunt trauma to a kidney in a patient with Autosomal Dominant Polycystic Kidney Disease, and to summarize the relevant literature reporting on similar cases. Despite the presence of well-established protocols for the diagnosis and management of blunt renal trauma, managing emergencies with trauma to a kidney in patients with ADPKD is not common. This review will hopefully be an educational opportunity in the diagnosis and management of blunt trauma to kidneys in patients with ADPKD. We sought to add to the growing literature by focusing more on cases with ADPKD and describing an uncommon type of renal injury that was managed differently from the aforementioned cases.

### Literature review

A literature review of the published cases of ADPKD kidneys that presented with blunt trauma was performed by searching the following databases: EMBASE, PubMed, Google Scholar, and Medline. Search terms included: “Abnormal Kidneys”, “Pathologic Kidneys”, “Polycystic Kidney”, “Autosomal Dominant Polycystic Kidney Disease”, “Trauma”, “Blunt Trauma”, “Wounds and Injuries”, “Blunt Abdominal trauma”, “Blunt Renal Trauma”, “Blunt Renal Injury” “Pre-Existing Renal Lesions”, “Intra-abdominal bleeding”, “Intra-abdominal hemorrhage”, “Non-penetrating injury”.

The literature search revealed 12 published cases of trauma to pre-existing kidneys in patients with ADPKD. We suspect that the scarcity of case reports found in the literature is partly due to under-reporting. Table 1 summarizes the published cases found in the literature review according to demographics, mechanism of injury, presentation, CT abdomen findings, and treatment. The table serves as a quick reference of the currently available literature. The most common presenting complaint involved gross hematuria (7 cases) and abdominal/flank pain (7 cases). Abdominal CT was the diagnostic imaging of choice in all but one case and revealed injuries ranging from cyst rupture to American Association for the Surgery of Trauma (AAST) Grade IV injury to the kidney. Of the 12 cases, 4 cases required nephrectomy and 2 cases required selective embolization. Four of the 12 cases could be managed conservatively/non-operatively. Two of the 12 patients presented with unstable vital signs, notably a decreased blood pressure and an elevated heart rate, reflecting a state of shock. These patients were found to have ruptured cysts.

**Table 1:** Summary of cases of trauma to patients with polycystic kidney disease found in the literature

Authors	Age	Sex	Mechanism of injury	Presentation	CT abdomen findings	Treatment
Pandyan et al. [40]	55	F	Seatbelt contact during airplane rapid deceleration	Bilateral Flank pain + increased abdominal girth + gross hematuria	NR	Expectant
Gildenhuys et al. [23]	28	M	Assault by a brick	Flank pain + gross hematuria	Ruptured cyst	Bed rest and observation
Kim et al. [41]	39	F	Shock-wave lithotripsy	Shock	Ruptured cyst with peri-renal hematoma	Supportive care then nephrectomy
Klein et al. [31]	40	M	Assault by a baseball bat	Gross hematuria	Possible hemorrhagic cysts	Expectant
Leslie et al. [20]	22	M	Motor Vehicle Accident	Flank pain then abdominal pain + tachycardia	Bilateral ruptured hemorrhagic cysts	Bilateral nephrectomies
Mabillard et al. [24]	25	M	Rugby/contact sport injury	Flank pain + gross hematuria	Ruptured cyst + retroperitoneal hemorrhage	Embolization of lower polar segmental branch of renal artery
Mufarrij et al. [32]	77	M	Vigorous massage chair session	Right lower quadrant pain + right-sided hip pain then 2 syncopal episodes	Cystic hemorrhage and rupture	Expectant + blood transfusions
Nash et al. [21]	58	F	Motor Vehicle Accident	Gross hematuria + distended abdomen then hypovolemic shock	Ruptured cyst in right kidney + multiple hemorrhagic cysts in left kidney	Bilateral nephrectomies
Reay et al. [42]	20	M	Fall from a horse with elbow flexed into flank	Gross hematuria	Renal injury into pelvicalyceal system and perinephric hematoma	Coil embolization of bleeding renal artery and pseudo aneurysm
Rhyner et al. [43]	NR	NR	Unknown	Unknown	Intracystic + retroperitoneal hemorrhage	Unknown
Wani et al. [33]	56	M	Hit by a thick rope on the flank	Flank pain + gross hematuria	Intracystic hemorrhage	Expectant
Zaslau et al. [44]	29	M	Blunt trauma to flank from ladder Fall of 8 ft in height	Flank pain + drop in hematocrit	Retroperitoneal hematoma and fluid + ruptured cyst	Nephrectomy

*F* female, *CT* computed tomography

*M* male, *NR* not reported

## Case presentation

This is the case of a 75-year-old male with a history of ADPKD, hypertension, dyslipidemia, Crohn’s disease, Benign Prostatic Hyperplasia and a left nephrectomy who presented with generalized malaise, mild diaphoresis, right lower quadrant abdominal pain 10 min after tripping and falling on an outstretched arm while walking down the street. Immediately after the fall, he complained of right wrist pain, walked a short distance back home and started complaining of malaise. He was subsequently evaluated by a neighbor Emergency Medicine attending physician who suspected hypovolemic shock, and transported him in the back of a car to a nearby tertiary care Emergency Department (ED). In route to the hospital, he became pre-syncopal, improving on Trendelenburg positioning. Upon arrival to the ED, his vital signs were as follows:

Temperature = 36.2 (degrees Celsius), Blood Pressure: 100/62 mmHg (130/72 Trendelenberg), Heart Rate = 53 beats/min, Oxygen saturation = 100%, Respiratory Rate = 27 breaths/min.

On physical exam, he was profusely diaphoretic, lethargic but alert and oriented to person, place and time. He had right lower quadrant abdominal tenderness with no rebound or guarding. Due to suspicion of shock, he immediately received two boluses of 0.9% Normal Saline (1 l each). Next, 2 units of O negative packed Red Blood Cells were prepared and he was immediately transfused. The patient was not receiving anticoagulation, so reversing the latter was not envisioned. He reported that his baseline creatinine is 1.6 mg/dL.

His home medications included Irbesartan, Budesonide, Fenofibrate, Ezetimibe, Allopurinol, Nebivolol, Calcium Carbonate and Finasteride.

**Fig. 1:**
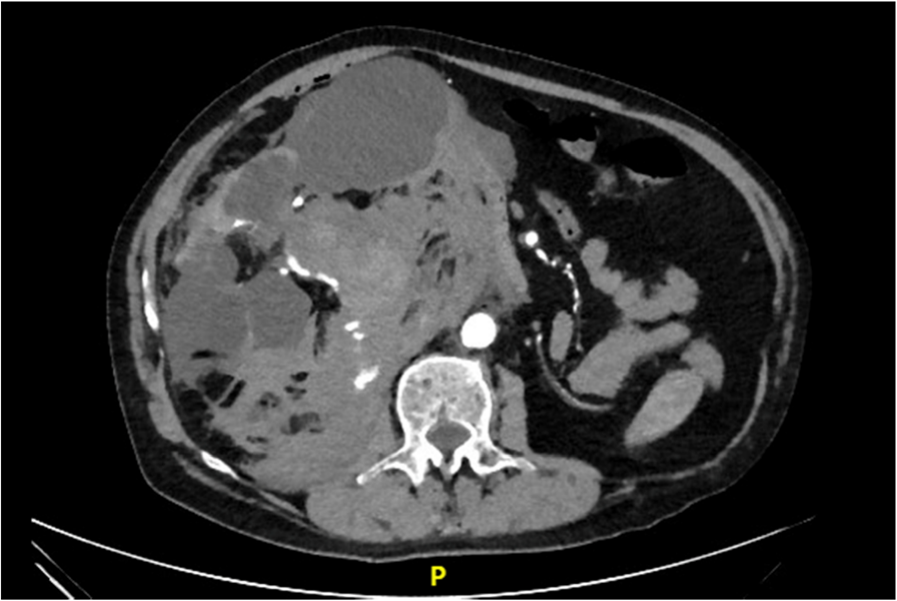
Axial CT scan of the abdomen and pelvis showing active contrast extravasation along the posterior aspect of a right perinephric hematoma extending into its most dependent aspect on the arterial phase associated with contrast pooling- consistent with an acute bleed; most likely arising from a right lower segment renal artery

**Fig. 2:**
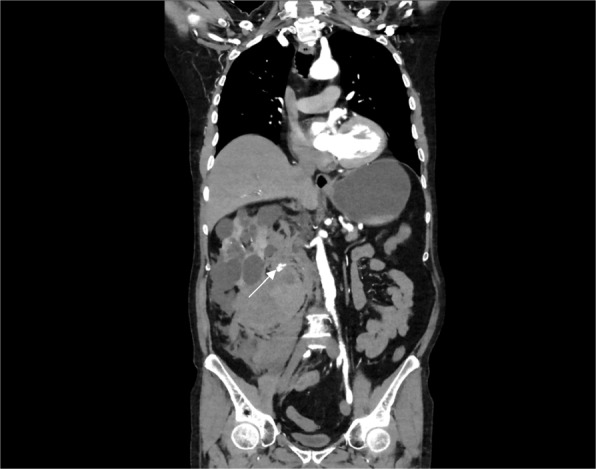
Reformatted Coronal CT scan image demonstrating patency of the main renal artery and area of extravasation involving the lower pole region of the polycystic kidney (denoted by a white arrow)

The risk/benefit ratio for contrast administration was evaluated and decision was made to obtain a CT angiography of the abdomen and pelvis, which revealed the following:

- A large hematoma measuring 11 × 7 × 7.5 cm inseparable from the medial aspect of the right lower renal pole and extending into the right perinephric space, displacing the kidney superiorly and anteriorly; with associated significant perinephric blood and associated minimal retroperitoneal bleed.
- Active contrast extravasation along the posterior aspect of the hematoma extending into its most dependent aspect (Figs. 1, 2) on the arterial phase associated with contrast pooling on the venous and delayed images consistent with an acute bleed; most likely arising from a right lower segment renal artery.
- A 12-min delay image demonstrated pelvic contrast extravasation from the proximal ureter, indicating urinary extravasation (Additional file 1).

In summary, the CT was in keeping with an acute right renal bleed likely arising from a right lower segmental artery, with an associated large right perinephric hematoma.

Laboratory results revealed a hemoglobin of 13.3 g/dL, white cell count of 9700 /cu.mm, a platelet count of 237,000 /cu.mm, an INR of 1, a lactic acid of 3.86 mmol/L, Creatinine of 1.9 mg/dL, and a GFR of 34 mL/min/1.73m^2^.

Urine dipstick revealed 2+ protein, 4+ qualitative hemoglobin, numerous RBCs and 8–10 WBCs.

After an urgent consultation with a multidisciplinary team including Urology, Vascular Surgery, Trauma Surgery and Interventional Radiology, a decision was made to proceed with a supra-selective right renal artery angiography and segmental artery embolization.

**Fig. 3:**
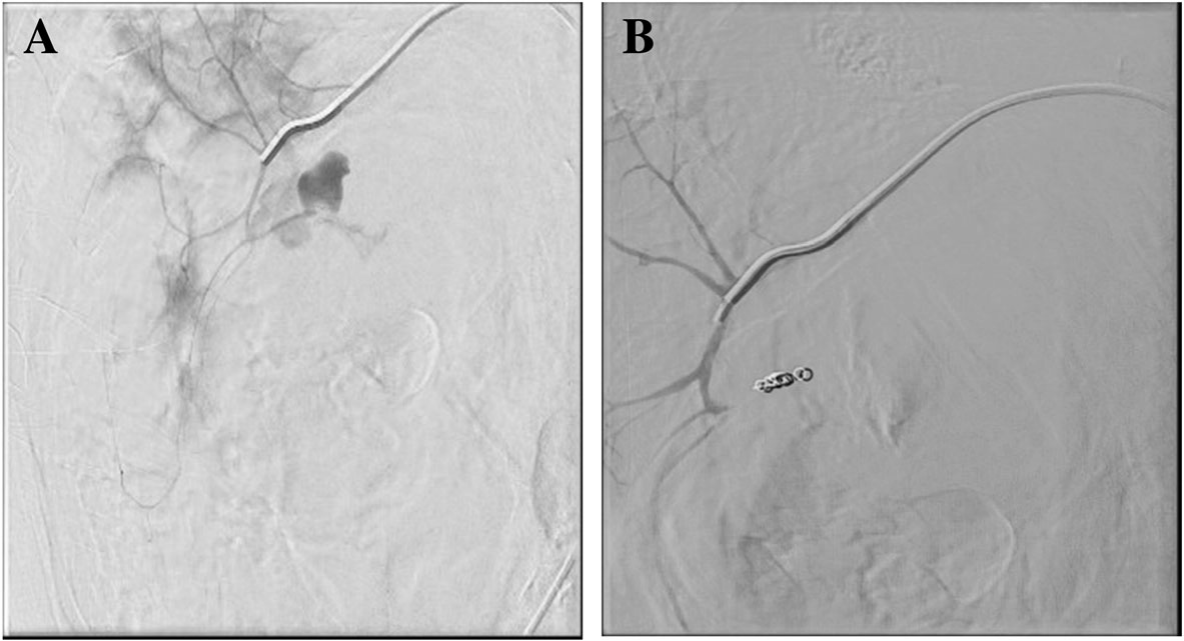
**a**. Selective catheterization of the inferior segmental branch of the right renal artery showing active extravasation and pooling of contrast at the lower pole branch of the right renal artery with opacification of one of the cysts. **b**. Completion selective angiogram showing interruption of flow (no contrast extravasation) after deployment of 2 coils

Successful and uneventful embolization of the active bleed of the lower pole branch of the right renal artery was performed by the interventional radiology team (Fig. 3). The patient tolerated the procedure well and left the interventional unit in a stable condition. He was subsequently admitted to the surgical ICU for monitoring, IV hydration, blood transfusion and serial Creatinine level checking.

The patient subsequently developed an acute kidney injury with a Creatinine reaching 2.8 mg/dL the day after the procedure. The same day, repeated Hemoglobin levels revealed a Hemoglobin of 10.3 g/dL that dropped further 2 days later reaching a trough of 7.3 g/dL. The patient received a total of 3 units of packed RBCs during his hospital stay.

The patient was discharged 6 days later after improvement in his Hemoglobin levels and hemodynamic stabilization. His Creatinine on discharge was 1.5 mg/dL and his Hemoglobin was 9.0 g/dL. Four days after discharge the patient remained stable clinically with a Hemoglobin of 9.7 g/dL and Creatinine of 1.6 mg/dL. Follow up with CT imaging 5 days after discharge did not show any increase in peri-nephric hematoma to indicate continuous bleed or failure of embolization.

## Discussion and conclusions

This paper reports on a patient with ADPKD who exhibited rapid clinical deterioration after minimal trauma, and compares the case to other similar cases reported in the literature. The main motives behind writing this case report were as follows:

- Shed the light on the importance of suspecting renal injury in patients with pre-existing renal lesions (PRELs) irrespective of the mechanism of injury; and, vice-versa to suspect a PREL in patients with a clinical deterioration that’s out of proportion to the mechanism of injury.
- Report on the uncommon injury pattern observed in our patient, and its management; along with the available management options.
- Highlight the importance of maintaining a high index of suspicion for intra-abdominal injury and bleeding in elderly patients, irrespective of anticoagulant use.

The kidneys are the most frequently injured genitourinary organ secondary to trauma. Renal injury occurs in up to 5% of trauma victims, and is responsible for 24% of traumatic abdominal solid organ injuries [7–9]. The kidney is particularly susceptible to deceleration injuries (such as falls and motor vehicle collisions) because it is spatially fixed merely by the renal pelvis and the vascular pedicle. Pre-existing renal lesions increase the risk of injury to the kidneys after blunt abdominal trauma [10]. Specifically, the risk of hemorrhage is amplified with increasing kidney volume, particularly if the kidneys are greater than 15 cm in length or if there is hypertension or renal impairment [11, 12].

ADPKD occurs in all races and has a described prevalence of 1:400 to 1:1000 [13]. Virtually all individuals who inherit the *PKD1* or *PKD2* genes eventually develop renal cysts that are visible by ultrasonography and cause enlargement of the kidneys [14]. Patients with ADPKD usually present with hypertension, hematuria, proteinuria, or renal insufficiency, discovered on routine laboratory examinations. Flank pain due to hemorrhage, urinary tract infection or stones are the most common symptoms reported by patients [15].

A study by Brown et al. showed that in trauma patients, older age was an independent risk factor for mortality for the overall population and across all mechanisms. Falls were also shown to be the most common mechanism for geriatric trauma patients [16]. It is therefore important to thoroughly evaluate elderly trauma patients.

In a study by Schmidlin et al. investigating injury biomechanics on a computerized kidney model, maximal stress concentrations were found to be at the periphery (outer surface of the kidney). This study also illustrated how the presence of a liquid-filled cyst causes stress concentration by amplifying the concept of impact force in the setting of blunt trauma, and weakening of the model as a whole [17]. These findings potentially validate why most of the patients in this review suffered from ruptured cysts. Injuring the renal vasculature, which resides in the kidney pedicle (centrally) is thus not a common occurrence and an alternative biomechanical explanation must be provided [18].

However, deceleration or acceleration type of blunt trauma are the most common causes of renal pedicle injuries; including rupture or thrombosis of the main renal artery, branch of renal artery, or renal vein [19]. Our patient fell on an outstretched hand from a standing height while walking. We suspect that upon sudden deceleration, the weight of the polycystic kidney must have caused an avulsion of the renal pedicle, thus lacerating the lower segmental branch.

Two other studies report on patients that injured the renal vasculature. One mechanism is injury during a rugby game (Mabillard et al.) and the other is a fall from a horse with the elbow flexed into the flank. The former resulted in a ruptured cyst and the latter in a pelvicalyceal injury, as found on the CT abdomen [20, 21]. Interestingly, the patient reported by Mabillard et al. injured his lower segmental renal artery branch just like our patient, despite having a different mechanism of injury (contact sport).

Our patient presented with a clinical picture suggestive of early hypovolemic shock, warranting urgent investigation and stabilization. The risks of contrast-induced nephropathy in this hypovolemic patient with a single kidney were weighed against the benefits of establishing the rapid diagnosis, and he was thus rushed to CT scanning. In fact, a recently published review by Luk et al. [22] emphasized that contrast-induced nephropathy risk is often overestimated and described how refraining from using contrast can cause misdiagnosis and delay in proper patient management.

To note, none of the patients who suffered from a vascular injury presented with a state of shock. In our literature review, the patients that displayed a clinical picture of shock had the following mechanisms of injury: shock-wave lithotripsy and a vigorous massage chair session [23, 24]. Our patient presented with hypovolemic shock following a fall on an outstretched hand from a standing height. This suggest a poor correlation between the mechanism of injury and the severity of the clinical presentation in patients with pre-existing renal lesions.

Furthermore, renal imaging is necessary in blunt injuries whenever patients have gross hematuria or microscopic hematuria (≥3 to 5 RBCs/HPF) in the presence of shock. Additional relative indications include a major decelerating mechanism (e.g. high-speed MVC or a fall from height) and clinical evidence of renal injury (such as flank bruising or tenderness) [25, 26]. The imaging modality of choice is CT scanning with intravenous contrast [27].

The need for surgical repair of renal trauma depends on how severe the injury is, as classified by the AAST organ injury severity scale for the kidney (Table 2) [27]. Most grade I and II injuries can be treated non-operatively whereas grade V injuries often necessitate nephrectomy, which can be life-saving in the rare occurrence of exsanguinating injuries. Since the CT scan revealed right renal bleed likely arising from a right lower segmental artery, the injury classifies as a grade IV vascular injury, the definitive management of which is a subject of debate. In fact, treatment options vary, and depend on hemodynamic status and associated injuries. These include operative angioembolization, nephrectomy, or observation [28]. When there is major renal vascular disruption, urgent angiography with selective embolization can be both therapeutic and diagnostic. Nevertheless, angiography requires a lot of resources, including significant time, specialized equipment and expertise which is not available in all medical centers, at all times. Given that our patient had a single kidney, did not respond optimally to hemodynamic support (2 transfusions and fluid resuscitation) and given the availability of an expert interventional radiology team, the medical team decided that angio-embolization would be the best fit for this patient.

**Table 2:** American Association for the Surgery of Trauma organ injury severity scale for the kidney

Grade	Type	Description
I	Contusion	Microscopic or gross hematuria, urologic studies normal
	Hematoma	Subcapsular, nonexpanding hematoma without parenchymal laceration
II	Hematoma	Non-expanding peri-renal hematoma confined to renal retroperitoneum
	Laceration	Laceration < 1 cm depth of renal cortex without urinary extravasation
III	Laceration	Laceration > 1 cm depth of renal cortex without collecting system rupture or urinary extravasation
IV	Laceration	Parenchymal laceration extending through renal cortex, medulla, and collecting system
	Vascular	Main renal artery or vein injury with contained hemorrhage
V	Laceration	Completely shattered kidney
	Vascular	Avulsion of renal hilum, devascularizing the kidney

*Santucci RA, McAninch JW, Safir M,* et al. *Validation of the American Association for the Surgery of Trauma organ injury severity scale for the kidney. J Trauma 2001; 50:195. Copyright © 2001 Lippincott Williams & Wilkins*

In the presence of renal vasculature injury, multiple severe associated injuries can be present most of the time, with a mortality rate reaching 44%. Despite urgent radiologic evaluation leading to rapid diagnosis, most patients are usually not candidates for vascular repair because of the frequent incidence of severe associated injuries to the same kidney. In spite of vascular repair, kidney function does not usually return to normal [19]. In addition, reversal of anticoagulation should always be considered, as it complements controlling the source of bleeding [29]. Desmopressin, a vasopressin analog, is a hemostatic agent that stimulates the release of von Willebrand factor from endothelial cells. Its use has been well-recognized in uremia-induced platelet dysfunction and should be considered as a treatment modality in uremic bleeding [30].

All the cases that underwent nephrectomies, had CT abdomen findings of ruptured cysts [23, 31–33]. However, the opposite does not hold true. A radiological diagnosis of ruptured cyst was therefore not sufficient to warrant nephrectomy, in the cases presented. In fact, in a study that examined 8465 trauma patients with renal injuries, 4% of whom required nephrectomies, the strongest risk factor for nephrectomy was found to be the severity of renal injury according to the AAST organ injury scale for Renal Trauma [34]. On the other hand, in a retrospective review by Altman et al., it was shown that conservative management of blunt grade 5 renal injury is feasible in patients who are hemodynamically stable at presentation [35].

In addition, several studies validate the importance of the AAST organ injury scale but have also found that perinephric hematoma size and contrast extravasation were independently associated with urological intervention [36, 37], both of which were seen in the patient described in this case. Two studies specifically showed that a hematoma greater than 3.5 cm was predictive of intervention [37, 38]. It is therefore prudent to always take into consideration the patient’s presentation, size of hematoma and grade of injury to help in the decision-making process.

Finally, follow-up CT imaging (after 48 h) is judicious in patients with deep renal injuries (AAST Grade IV-V) because they are prone to developing troublesome complications such as urinomas or hemorrhage [38, 39]. Of note, periodic monitoring of blood pressure up to a year after the injury could potentially uncover the rare instances of post-injury renovascular hypertension.

## Conclusion

Cases of traumatic injuries in patients with polycystic disease are a rare occurrence and therefore management guidelines are still not established. Further studies and expert consensus opinions are required to come up with recommendations on optimal management of this vulnerable patient population. Currently the majority of patients are managed according to the underlying abnormality and injuries, such as described in the above case. Rapid clinical deterioration after minimal trauma should raise suspicion of an underlying abnormality. In the right circumstances, selective renal artery embolization is a safe option in patients who sustain a renovascular injury.

## Additional file

Additional file 1: Axial CT scan 12-min delay image demonstrating urine extravasation from the proximal ureter (highlighted by the white arrow). (DOCX 761 kb)

## Abbreviations

AAST: American Association for the Surgery of Trauma
ADPKD: Autosomal Dominant Polycystic Kidney Disease
cm: Centimeter
CT: Computed Tomography
cu.mm: Cubic Millimeter
dL: Deciliter
ED: Emergency Department
F: Female
g: Gram
HPF: High Power Field
ICU: Intensive Care Unit
IV: Intravenous
L: Liter
M: Male
m^2^: Meter Squared
mg: Milligram
min: minute
mmHg: Millimeters of Mercury
mmol: Millimoles
MVA: Motor Vehicle Accident
NR: Not Reported
PCKD: Polycystic Kidney Disease
PREL: Pre-existing Renal Lesion
RBC: Red Blood Cell
