# Supplementary figures and images for: Autosomal dominant polycystic kidney disease and minimal trauma: medical review and case report
Source: BMC Emerg Med. 2018 Nov 1;18:38. doi: 10.1186/s12873-018-0192-3 (PMC6211544; doi:10.1186/s12873-018-0192-3)

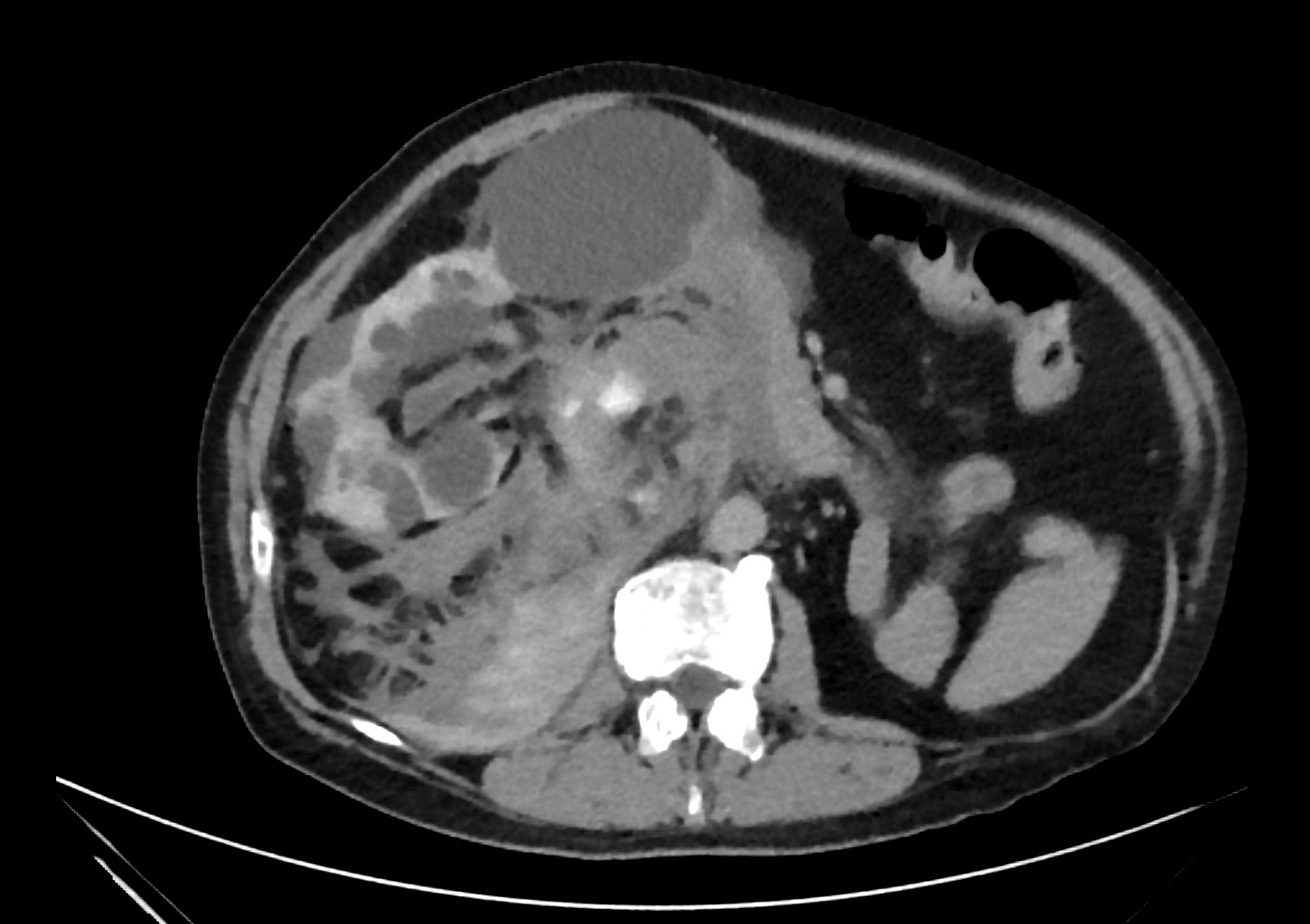

Supplement: Supplementary file 1 — Axial CT scan 12-min delay image demonstrating urine extravasation from the proximal ureter (highlighted by the white arrow). (DOCX 761 kb) [file 12873_2018_192_MOESM1_ESM.docx]
